# Revisiting the role of pollen-microbiome interactions: new insights into the “One Health-One Biosecurity” concept in changing agroecosystems

**DOI:** 10.3389/fmicb.2025.1620446

**Published:** 2025-08-01

**Authors:** Jakab Máté Scherman, Gábor Markó, Erzsébet Szathmáry, Géza Nagy, Ivett Kocsis, Marietta Petróczy

**Affiliations:** Department of Plant Pathology, Institute of Plant Protection, Hungarian University of Agriculture and Life Sciences, Budapest, Hungary

**Keywords:** pollen, natural pollen-spore trap, pathogenesis, germination, microbiome, phyllosphere

## 1 Introduction

The One Health-One Biosecurity framework recognizes the interconnectedness of human, animal, plant, and environmental health (Hulme, 2020). This concept can easily be illustrated through plant diseases caused by phytopathogens. For example, the cereal crop pathogen *Fusarium graminearum* principally infects hosts through floral tissues (Khalaf et al., 2021), resulting in significant yield losses and contamination of grains with mycotoxins like deoxynivalenol. This toxin not only threatens plant health and agricultural productivity but also poses serious food and feed safety risks for humans and animals, causing gastrointestinal symptoms, feed refusal, and broader public health concerns (Patriarca and Fernández Pinto, 2017). The pathogenesis is influenced by several major factors shaped by many contributing subfactors that may be relevant in the context of increasing pathogen pressure in agroecosystems. One such element is the presence of pollen grains, whose specific role is underrepresented.

Pollen is widely recognized as essential for plant sexual reproduction, promoting genetic and phenotypic diversity among offspring (Hafidh and Honys, 2021). However, its ecological role extends far beyond pollination. Plant surfaces (e.g., leaves, bark) naturally accumulate airborne particles as passive traps, capturing pollen grains (Faegri et al., 1989; Groenman-van Waateringe, 1998; Zhang et al., 2020) and fungal spores (Magyar, 2008).

Increasing evidence suggests that pollen grains and their water-soluble exudates serve as primary and supplementary nutrient sources for microbes. These humid, nutrient-rich microhabitats promote spore germination, growth, and infection of several pathogenic and non-pathogenic fungi (Hennebert, 1973; Huang et al., 1998). The presence of pollen increases the virulence of plant pathogens (Fourie and Holz, 1998), which is crucial in the early stages of fungal development and pathogenesis (Chou and Preece, 1968; Allen et al., 1983).

Early experiments revealed a pollen-driven stimulatory effect on certain host plant–pathogen interactions (Bachelder and Orton, 1962; Chou and Preece, 1968). Recent research has shown that this phenomenon may extend beyond host specificity, indicating a broader ecological mechanism (Kocsis et al., 2022). Nevertheless, the mechanisms underlying pollen-stimulated spore germination, species-specific variations, and microbiome-driven effects remain poorly understood.

Notably, pollen grains are not sterile; they harbor diverse microbial communities, including bacteria (Manirajan et al., 2018), fungi (Naggar and Sallam, 2009), and viruses (Fetters and Ashman, 2023). These microorganisms along with airborne pollen, spores, and other pollutants may influence the structure and function of phyllosphere microbiota (Annamalai and Namasivayam, 2015; Leveau, 2019). In human-altered ecosystems, such as agricultural fields, a diverse microbiome has been shown to improve plant health, thereby enhancing disease resistance (Berg and Koskella, 2018; Perreault and Laforest-Lapointe, 2022).

Therefore, this opinion article aims to review the current evidence on the role of pollen-associated microbiota in plant–pathogen interactions, explore its relevance within the One Health-One Biosecurity framework, and identify critical research gaps while suggesting future research targets essential for advancing agricultural production and crop protection.

## 2 Sources, transport and accumulation of pollen and pathogen spores and the stimulatory effect

Pollen disperses via wind or pollinators over varying distances (Wessinger, 2021; Rodrigues et al., 2023). Most airborne pollen originates from anemophilous plants, and the dispersal patterns vary according to flowering periods (Jones and Harrison, 2004; Wozniak and Steiner, 2017), atmospheric conditions (Raynor et al., 1974), unique pollen morphology (e.g., *Ambrosia*: spiked structure; *Pinus*: air-filled bladders), and water content (Schwendemann et al., 2007; Sabban and van Hout, 2011). Wind-pollinated crops, such as corn (*Zea mays*) and sugarcane (*Saccharum officinarum*), dominate global grain and sugar production (Klein et al., 2007), and common wind-pollinated weed species, including *Artemisia* and *Rumex* genera, or the *Poaceae* and Urticaceae families, contribute significantly to airborne pollen loads (Bogawski et al., 2014).

Fungal aerosols also arise from vegetation, with plant leaf surfaces serving as key sources of airborne propagules (Awad, 2005; Qi et al., 2020). Agricultural systems harbor crop-specific pathogenic fungi that produce large quantities of spores (Obayori, 2023). The most prevalent airborne fungal genera from agriculture include *Alternaria, Cladosporium, Penicillium, Aspergillus*, and *Fusarium* (Al-Shaarani and Pecoraro, 2024). The vast majority of plant pathogens are airborne (Fagade et al., 2023), and their spore production and dispersal are influenced by weather, geography, and human activities (Cho et al., 2006). Thus, natural habitats and agricultural landscapes are primary and major sources of airborne pollen grains and fungal spores.

Beyond wind-mediated dispersal, insect pollinators play a significant role in pollen transport and plant fertilization. *Hymenoptera* and *Lepidoptera* species are the primary vectors (Primack and Silander, 1975; Schemske and Horvitz, 1984; Herrera, 1987). Over one-third of agricultural crops depend on insect pollination (Klein et al., 2007), including key crops, such as oil-palm (*Elaeis guineensis*), soybean (*Glycine max*), sunflower (*Helianthus annuus*), and rapeseed (*Brassica napus*), which are essential contributors to global food systems (Roubik, 2018).

Pollinators interact with numerous flowers during feeding, acting as microbial vectors and mixing pollen (Brett, 1966) and related microbiota across plant species. The activity of pollinator species, and the floral abundance can directly or indirectly shape microbial communities (Wei et al., 2021). For example, honeybee behavior increases bacterial diversity, introducing symbionts, bee pathogens, and nectar-associated microbes (Prado et al., 2022). Interestingly, insect-pollinated species may host microbiomes more similar to wind-pollinated species, potentially due to this microbial exchange (Ambika Manirajan et al., 2016). Honeybees also spread the flower pathogen *Erwinia amylovora* while foraging (Cellini et al., 2019). Other pseudo-flower and flower-scent-producing pathogens, such as *Puccinia* spp., *Microbotryum violaceum*, or *Monilinia vaccinii*-*corymbosi*, are also pollinator-dispersed (Raguso and Roy, 1998; Dötterl et al., 2009; McArt et al., 2016). Similarly, honeybees can also disseminate fungal spores incidentally collected from insect honeydew or plant surfaces (Shaw, 2015).

Interactions between pollen and microbes can occur at different levels, such as in the atmosphere and in the phyllosphere. Pollen grains can absorb up to 100% of their weight in water under humid conditions (Diehl et al., 2001). Due to their large size and hygroscopic properties, pollen grains, together with airborne spores, play a role in cloud formation as cloud condensation nuclei (Ariya and Amyot, 2004). The extraction of pollen grains and mobilization of nutrients could start in this aqueous environment. The water-soluble fraction of cloud vapor from pollen grains contains sugars (e.g., fructose, glucose, sucrose, trehalose) and sugar alcohols (e.g., arabitol, inositol, mannitol), which, when falling with the precipitation, create a nutrient-rich and stimulating microenvironment for spore germination (Wang et al., 2007; Yttri et al., 2007; Hayer et al., 2013).

At the phyllosphere level, the accumulation of pollen grains provides nutrients and chemical trigger molecules for fungal spores. The rapid biological exploitation of pollen resources by both parasitic and saprophytic microorganisms is evidence based. For example, pollen grains of corn have been shown to stimulate the early germination of macroconidia spores in *Fusarium* species, which cause serious stem rot (Naik and Busch, 1978). The presence of pollen stimulates spore germination of *Botrytis cinerea* as well (Chou and Preece, 1968; Kocsis et al., 2022). For litter- and wood-decaying fungi, including members of the Basidiomycota (Hutchison and Barron, 1997) and Phycomycetes (Goldstein, 1960), pollen provides a supplementary seasonal source of nutrients. This phenomenon is a general stimulatory effect, as the initial development of many fungal species can be widely triggered by pollen extracts. Furthermore, it has been demonstrated that this effect is not plant species-specific, because pollen grains of numerous plant species exert an influence on the same fungal species (Kocsis et al., 2022).

Droplets containing pollen can also harbor bacteria that affect fungal spore germination. Bacteria are often observed surrounding fungal spores, such as *Botrytis cinerea*, on leaf surfaces, where they inhibit spore germination by depleting available nutrients (Blakeman, 1973). Recent research has uncovered that maize pollen harbors beneficial bacteria capable of suppressing fungal pathogens, highlighting pollen's role beyond fertilization (Shrestha et al., 2024). This discovery emphasizes the importance of considering pollen as a dynamic microenvironment influencing plant–microbe interactions and pathogen dynamics. Studies have shown that the presence of pollen increases the performance of beneficial microorganisms used in biological control strategies (Li et al., 2003). Thus, although pollen generally stimulates pathogen spore germination by enriching the microenvironment, it can also enhance the effectiveness of biological control agents.

## 3 Discussion and future perspectives

In the last two decades, the microbiome concept has revolutionized our understanding of organism–environment interactions, particularly through the lens of ecological network theory (Foster et al., 2008). Recently, attention has turned toward the interactions between pollen and its associated microbiome, opening new perspectives in plant ecology, health, and crop production.

The impact of pollen-associated microbiomes on reproductive success (i.e., crop yield and quality) and long-term fitness consequences is crucial for plants living in natural or agricultural environments (Zasloff, 2017). Future research should prioritize understanding how microbial colonization interferes with pollen-pistil compatibility and signaling pathways, including signaling proteins and the sensitivity of recognition receptors, and how these factors affect fertilization efficiency, post-pollination processes (i.e., pollen tube growth or stigma health), seed set, and fruit development. Moreover, pollen microbiomes may be transmitted to seeds (Wu et al., 2022; Cardinale and Schnell, 2024), raising important questions about the inheritance of beneficial microbiomes and their role in plant fitness. Changes in pollen microbiome composition, which are associated with phylogenetic variation, contribute to host diversification (Khalaf et al., 2023).

The pollen microbiome composition is taxon-specific (Armstrong et al., 2024). Therefore, a thorough examination of the role of pollination type, pollinator, and wind-mediated transfer in dispersing both beneficial and pathogenic microbes is imperative. Understanding animal behavioral aspects (i.e., flower preference and feeding), as well as the implications for animal health and honey production, is crucial. In a broader context, the transfer of pollen may influence evolutionary processes through horizontal gene transfer, enabling gene flow between microbial communities across ecosystems. Pollen grains and fungal spores, notably those of the *Alternaria* species, have been observed to be transferred together (Magyar et al., 2022). This co-transfer provides an opportunity for gene transfer between different *Alternaria alternata* strains, a phenomenon that has already been documented (Mehrabi et al., 2011). These processes are especially important in agroecosystems, where pollen-associated microbes may transfer resistance genes due to regular chemical interventions.

The largest barrier to microbiome profiling using metagenomics is the limited accessibility of these methods due to high costs and the challenges associated with processing small amounts of DNA. However, ongoing methodological advances will enable more detailed and large-scale studies of pollen-associated microbial networks, ultimately supporting the development of greener, more resilient agricultural systems.

**Figure 1:**
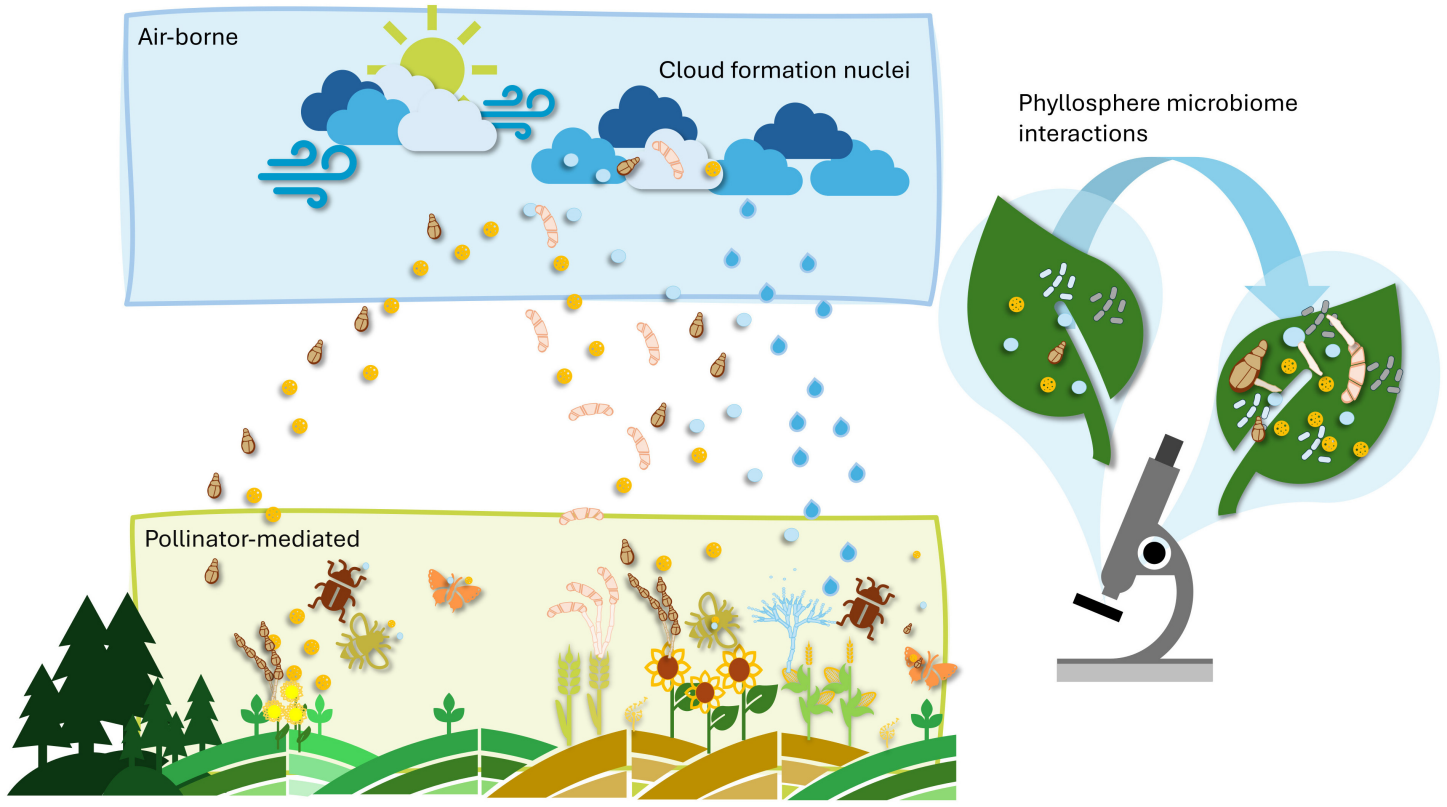
Airborne and pollinator-mediated dispersal of pollen grains and microbes shaping phyllosphere communities.

Human-related environmental factors, such as agricultural practices, pollution, and urbanization modify communities (Obersteiner et al., 2016), and reduce microbial richness, potentially weakening the natural defense systems of crops. In plant protection strategies, protecting not only pollinators but also the microbial communities associated with crops should become a priority. Non-selective chemical pesticides can harm not only targeted pathogens but also beneficial microorganisms within the microbial community, leading to dysbiosis (i.e., an imbalance in the microbiome; Iebba et al., 2016). This can increase a plant's vulnerability to future pathogen infections and abiotic stresses. Therefore, after chemical treatments, the restoration of the microbiome could minimize microbial gaps that leave plants vulnerable to infection. Given that pollen extracts can stimulate fungal germination, they may also be harnessed to encourage the growth of beneficial microorganisms (Figure 1), offering a novel biocontrol strategy after considering the associated risks to the environment and human health. Moreover, a highly diverse production environment (e.g., cover crops), combined with the development of microbiome-friendly agricultural practices, promotes microbial abundance on pollen and plant surfaces.

As final remarks, pollen-microbiome interactions are complex and crucial for plant health and agricultural sustainability. It is essential to recognize and include the modifying effect of pollen in pathogen-phyllosphere and microbiome interactions, as this perspective is critical for advancing both research and practical applications in agriculture. Preserving the diversity of plant-associated microbiomes on the phyllosphere is vital for crop health and resilience. In line with the One Health-One Biosecurity framework, future agricultural practices should focus on supporting microbiome-driven natural defenses rather than relying solely on pathogen control.
